# The interplay of socio-economic status represented by paternal educational level, white matter structure and reading

**DOI:** 10.1371/journal.pone.0215560

**Published:** 2019-05-02

**Authors:** Jolijn Vanderauwera, Ellie R. H. van Setten, Natasha M. Maurits, Ben A. M. Maassen

**Affiliations:** 1 Parenting and Special Education Research Unit, Faculty of Psychology and Educational Sciences, KU Leuven, Belgium; 2 Research Group ExpORL, Department of Neurosciences, KU Leuven, Belgium; 3 Center for Language and Cognition Groningen (CLCG), Faculty of Arts, University of Groningen, Groningen, the Netherlands; 4 Research School of Behavioural and Cognitive Neurosciences (BCN), University Medical Center Groningen, University of Groningen, Groningen, the Netherlands; 5 Department of Neurology, University Medical Center Groningen, University of Groningen, Groningen, the Netherlands; University of New Mexico, UNITED STATES

## Abstract

A child’s school achievement is influenced by environmental factors. The environmental factors, when represented by socio-economic status (SES) of the family, have been demonstrated to be related to the reading skills of a child. The neural correlates of the relation between SES and reading have been less thoroughly investigated. The present study expands current research by exploring the relation between SES, quantified by paternal educational level, reading of the offspring and the structure of white matter pathways in the left hemisphere as derived from DTI-based tractography analyses. Therefore, three dorsal white matter pathways, i.e. the long, anterior and posterior segments of the arcuate fasciculus (AF), and three ventral white matter pathways, i.e. the inferior fronto-occipital fasciculus (IFOF), the inferior longitudinal fasciculus (ILF) and the uncinate fasciculus (UF), were manually dissected in the left hemisphere of 34 adolescents with a wide range of reading skills. The results demonstrated a relation between word reading, SES quantified by paternal educational level, and fractional anisotropy (FA) within the left dorsal AF segment and the left ventral UF. Thus, the present study proposes a relationship between paternal educational level and a specific white matter pathway that is important for reading, aiming to guide future research that can determine processes underlying this relationship.

## Introduction

The environment in which a child grows up is known to substantially influence a child’s school achievement [1]. Parental characteristics are among the important factors influencing a child’s environment [2,3]. These parental-driven environmental characteristics can be represented by parental socio-economic status (SES). Quantification of SES is most commonly based on parental educational level, family income and parental occupation [4,5]. Lower socio-economic status has been related to poorer cognitive skills in children [6–8] as well as in adults [9,10], especially in the domains of executive functioning, language and memory [1,6,11–15]. Notably, a gap in reading achievement has been demonstrated in children with poorer SES [6,16–22], suggesting a link between reading and SES of the family in which the child grows up.

Acquisition of reading skills is demanding and requires formal instruction by teachers and/or caregivers. This is in contrast with language acquisition, which results from informal daily interaction between an infant and its parents and caregivers, considered to start from birth [23,24]. As our brain is not pre-determined to develop reading in evolutionary terms, during early years a child’s brain has to reorganize brain regions and connections, mainly involved in language, visual and auditory processing, to become literate [25]. Hence, the effortful process of learning to read is considered to result from a complex interplay between multiple factors, i.e. genes, the neurobiological system, cognitive skills, and environmental influences [26]. Understanding the interaction between all these factors is of crucial importance to get a comprehensive insight into the development of reading skills. This is especially important for those individuals developing dyslexia, a learning disability that affects 3–7% of the population [27] and is characterized by problems with accurate or fluent word recognition, poor decoding and poor spelling abilities [28]. A substantial number of studies has investigated the relation between cognitive factors and reading (impairment), and the neural correlates of these cognitive factors (for reviews and meta-analyses see [29–36]. Moreover, the relation between genes, reading and the brain has been investigated (e.g. [37–42]). However, while environmental influences, as quantified by SES, were acknowledged to be related to reading, it is largely unknown how these environmental influences interact with neural correlates of reading. To date, only one recent study investigated the direct relation between reading, specific white matter tracts of the reading network and SES. More specifically, the study by Ozenov-Palchik et al. [43] investigated this interplay in three white matter pathways, the long segment of the arcuate fasciculus, the SLF, here referred to as the anterior segment of the arcuate fasciculus, conforming to Catani & Thiebaut de Schotten [44] and the inferior longitudinal fasciculus (ILF). They reported a positive association between SES and fractional anisotropy (FA) in bilateral ILF. The investigation of long-range white matter pathways is of specific interest given that cortical regions of the reading network are widespread in the brain and the key role of white matter connections in reading and dyslexia has been widely argued [45–49]. In addition, two studies directly compared the relation between literacy, SES and structural brain measurements by means of whole-brain analyses. Jednoróg et al. [50] used a whole-brain method to study gray and white matter properties in a group of normal reading 8 to 10-year old children. They confirmed a relation between literacy, language skills and SES and revealed positive relations between gray matter volumes in bilateral hippocampi, left fusiform and right inferior occipito-temporal (OT) regions and SES. No relation between SES and white matter structure was found, as measured with tract-based spatial statistics (TBSS). Gullick et al. [51] applied a whole-brain voxel-wise method to investigate the relation between reading, SES and white matter structure in a sample of normal reading children aged 7 to 13 years old. They showed that the relationship between reading and FA in clusters attributed to the bilateral ventral inferior longitudinal fasciculus (ILF), left arcuate fasciculus (AF) and left corticospinal tract was mediated by SES, although interpretation of the results is difficult given that no direct relation between SES and reading was found.

Although only few studies directly unraveled the relation between environmental influences, reading and the brain, additional information can be derived from some studies that tried to define the neural correlates of SES. A few studies investigated how SES is related to brain functioning (for a review see [10]). SES has been shown to positively relate to left frontal cortex activation in 5-year old children during a rhyming task [52] and to resting state EEG activity in adolescents [53]. Noble et al. showed that the relation between phonological awareness and brain activity in reading-related areas, i.e. left fusiform and perisylvian regions, is modulated by SES [22]. SES has also been related to structural brain properties. Hanson et al. [54] investigated the relation between SES and hippocampal volume and reported a positive relation between SES and gray matter density measured by voxel-based morphometry (VBM) in bilateral hippocampi, although these results were not confirmed by Gianaros et al. [55]. The latter study did report a relation between gray matter volume in the anterior cingulate cortex and the subjective social status of adults. Chiang et al. [56] investigated the relation between SES and white matter fractional anisotropy (FA), by voxel-wise analyses. They reported no direct relations, although they did report genetic-environmental interactions between SES and genes affecting white matter properties.

**Fig 1:**
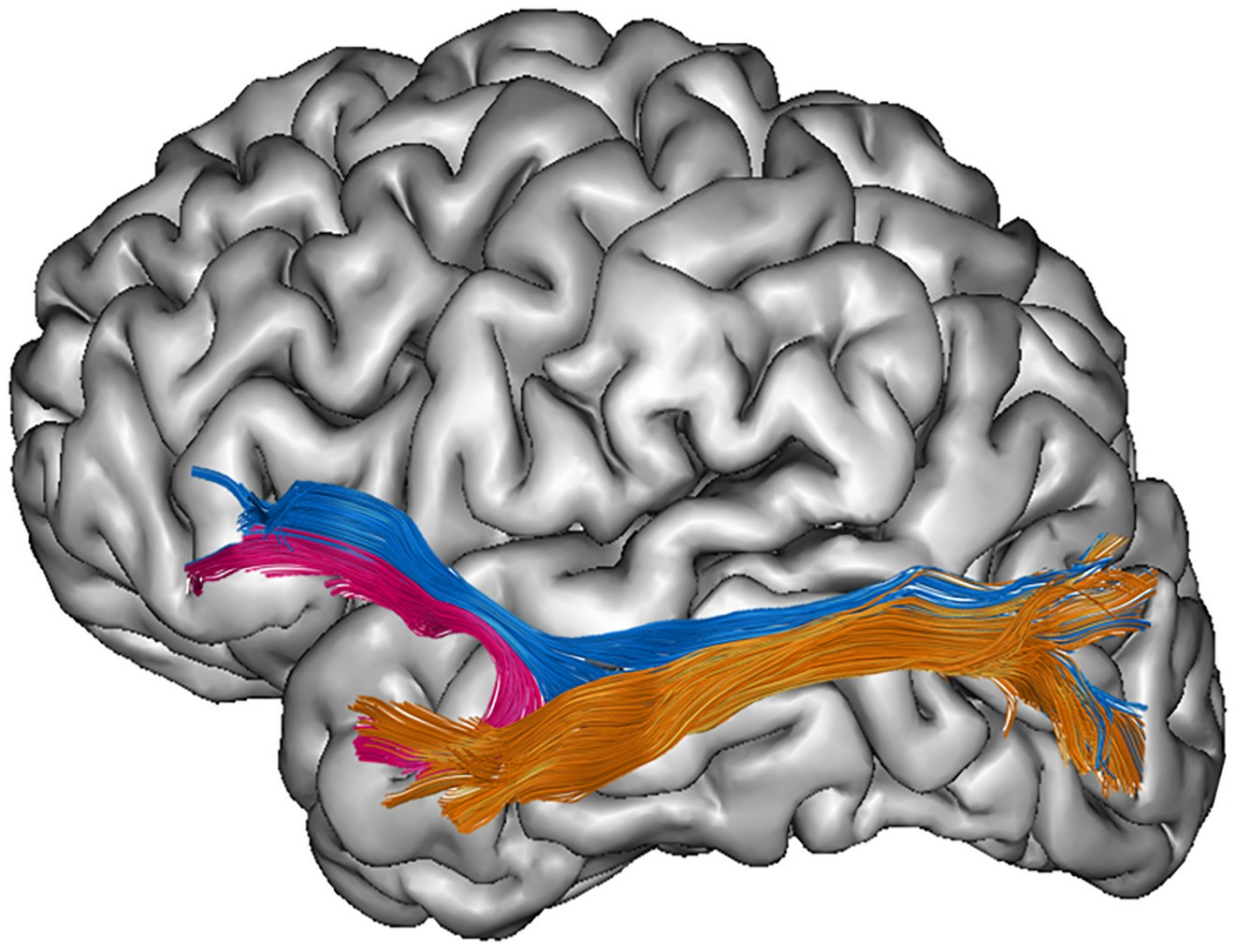
Visualization of the dorsal and ventral white matter pathways in the left hemisphere. In the left panel, the three dorsal segments of the arcuate fasciculus (AF) are presented, i.e. the long segment in *red*, the anterior segment in *yellow*, and the posterior segment in *green*. In the right panel, the three ventral white matter pathways are presented, i.e. the inferior fronto-occipital fasciculus (IFOF) in *blue*, the uncinate fasciculus (UF) in *pink* and the inferior longitudinal fasciculus in *orange*.

Although important insights result from these studies applying whole-brain approaches or investigating the neural correlates of SES, to date, the current study strives to extend the recent insights by Ozernov-Palchik et al. [43] on the relation between SES, reading and specific white matter pathways of the reading network. In experienced readers, especially the left hemisphere pathways are known to play a key role in the neuroanatomical reading network (for reviews see [45;49,57]). Hence, the present study aims to investigate the relationships between SES, reading skills and structural organization of left dorsal and ventral white matter pathways, for pathways that have previously been related to reading abilities in a sample of adolescents with broad ranging reading skills and socio-economic status. Understanding the role of all potential influences on the reading skills of a child is especially important for those children developing dyslexia. As the three dorsal segments of the arcuate fasciculus (AF), the ventral inferior fronto-occipital fasciculus (IFOF), the uncinate fasciculus (UF) and the inferior longitudinal fasciculus (ILF) have been related to reading processes and/or dyslexia, but with large variability in findings between studies, we manually dissected these six pathways in the left hemisphere to explore the potential role of SES on a substantial portion of the reading network. More specifically, virtual in vivo dissections of the three dorsal segments of the arcuate fasciculus (AF) and the ventral inferior fronto-occipital fasciculus (IFOF), the uncinate fasciculus (UF) and the inferior longitudinal fasciculus (ILF), were performed applying diffusion tensor imaging (DTI) tractography analyses (see Fig 1). Given that our study involves experienced readers, we focused on pathways in the left hemisphere. To investigate the specificity of the obtained results for SES, white matter and reading, we studied whether similar results were retrieved when reading was replaced by a broader language skill, namely vocabulary knowledge. Finally, having a family risk is known to be a significant risk factor for developing dyslexia, as it increases an individual’s chance to develop dyslexia up to 50% [58]. We investigated whether the results were (partly) driven by the family risk for dyslexia as an indirect proxy of the genetic factor.

## Methods

### Participants

In this study 35 adolescents aged 13 to 14 years old (Mean age = 13.7, SD = .5) participated. One adolescent was excluded because of an IQ below 80, resulting in a total participant sample of 34 adolescents. Fifteen participants were male and 19 were female, and all participants took part in the DDP (Dutch Dyslexia Program) in Groningen [59]. All adolescents attended regular Dutch secondary education, most were in grade 8, three in grade 7 and three in grade 9. All participants were native speakers of Dutch although some also spoke Frisian at home. The sample covers a broad range of reading skills. Seven participants were diagnosed with dyslexia; their word or pseudo-word reading fluency score, obtained using the same tests used in the present study that are described below, belonged to the lowest tenth percentile, while the score on the other test was also at least below average, on at least two out of three DDP measurements in grades 2, 3 and 6. For the present study we will use continuous reading scores in the analyses, as reading is a skill that follows a continuous distribution within the population [60], and developmental dyslexia is not an all-or-nothing disorder but rather is defined based on cut-off scores that are inherently arbitrary [31]. Eighteen participants had a high familial risk of dyslexia because they had at least one parent who reported him or herself as dyslexic, there was a self-reported family history of dyslexia, and the parent’s score on either word or pseudo-word reading fluency, was in the lowest twentieth percentile and on the other test at least in the lowest fortieth percentile using the norms by Kuijpers et al. [61]. For some participants only the dyslexic parent was tested, which is why the parental reading of adolescents with a high familial risk is not complete (see Table 1), but it was always verified that both parents of adolescents with a low familial risk did not meet any of the dyslexia criteria. Among the seven participants who were diagnosed with dyslexia, one participant had a low familial risk whereas six participants had a high familial risk. Similarl to the adolescent data, the continuous reading scores of the parents are used in our analyses instead of categories. Given the overrepresentation of adolescents with a family risk for dyslexia, the parents are expected to represent a broad variety of reading ability. For one participant the family risk status could not be determined because data were only available for one parent without dyslexia, and for another participant no valid reading classification could be made because due to missing data the persistence of the reading deficit could not be verified. These participants are included in the study as we do not use classifications. Parents had been recruited at midwife offices before their children were born. Participants with comorbid developmental disorders, as reported by the parents, were not excluded from the study as comorbidity between reading problems and developmental disorders is common. There were two participants with Attention Deficit Hyperactivity Disorder (ADHD), one with Attention Deficit Disorder (ADD) and one with Autism Spectrum Disorder (ASD). Note that excluding those participants from the analyses did not change the results. To go into the MRI scanner participants with metal in their bodies, claustrophobia or female participants that were pregnant could not be included. The medical ethical review board of the University Medical Center Groningen approved the study. Parents gave written informed consent and the adolescents gave informed assent for their participation in the study.

**Table 1:** Descriptive statistics of the adolescents and their fathers. Mean, standard deviation (SD), minimum and maximum scores are presented in the table. For each variable, the sample size (N) is presented.

	N	Mean (SD)	Range
*Adolescents*			
age (years)	34	13.7 (.5)	13.0–14.9
word reading score	34	79.1 (19.2)	22–102
pseudo-word reading score	34	73.6 (23.8)	10–116
vocabulary score	34	50.6 (8.9)	18–66
*Fathers*			
educational level (number of participants in category 1/2/3)	34	9/11/14	1–3

### Materials

#### Reading fluency

Reading skills of the adolescents were measured by word and pseudo-word reading fluency tests. When the adolescents were younger these same tasks were used at earlier DDP measurements. An A and B version of the tests were alternated to avoid a test-retest effect. Word reading fluency was assessed using the “Een-minuut-Test”; (one-minute test, [62]) and pseudo-word reading was tested using the “Klepel” test [63]. During these tests the participants had to read a list of words or pseudo-words, consisting of 116 items each, that increased in difficulty, as fast and accurately as possible within one minute for the word reading fluency test and within two minutes for the pseudo-word reading fluency test. The score is the number of words or pseudo-words read correctly.

#### Socio-economic status

Socio-economic status was quantified by parental educational level, conforming to Gullick et al. [51], as this measurement has been suggested to be the SES variable that best relates to reading [17] and is more stable than income or occupation [64]. At the start of the study, before the children were born, both parents indicated in a background questionnaire the highest level of education that they had achieved. Parental educational level was scored on a four-point scale from 1 to 4, where 1 is lower level secondary or vocational education, 2 is medium level secondary or vocational education, 3 is higher level secondary education and 4 higher level tertiary education including university level education and studies at universities of applied sciences. Note that for the fathers categories 2 and 3 have been combined, since only two fathers had a score of 3. Hence, a more balanced distribution within categories was obtained for the educational level of the fathers (category 1 = 9, category 2 = 11 and category 4 = 14). For the mothers, a less equal distribution was achieved since both categories 1 and 3 were relatively small (respectively one and three mothers) whereas category 4 contained 18 mothers. Moreover, data on the educational level of four mothers were missing. Therefore, we opted to further only include educational level of the fathers.

#### Vocabulary

Vocabulary knowledge was tested using the Dutch Wechsler Intelligence Scale for Children (WISC-III-NL, [65]). During this task the meanings of words with an increasing difficulty must be described. Per item two points can be gained for a fully correct item, and one point for a partially correct answer as described by the criteria in the manual. The test consists of 35 items in total. After 4 consecutive wrong answers the test was terminated.

### Procedure

The reading and vocabulary tests, which were part of a larger behavioral test battery, were usually conducted on a different day than the MRI scan, though not more than two weeks apart. If MRI examination and behavioral assessment had to be completed on the same day for practical reasons, there was a break of at least 2 hours between both measurements. Behavioral tests were conducted one-to-one in a quiet room and participants were instructed that they could take as many breaks as they wished. Together with the behavioral tests the adolescents were introduced to a dummy scanner and the MRI procedure was explained to them and to their parents. The DTI scans were part of a larger MRI protocol that lasted approximately one hour and included two fMRI tasks, as well. Both participants and parents were asked to rate how much anxiety the participant felt and how much they disliked going into the MRI scanner on a scale of 1–10. If scores were higher than 8 the participant could not take part in the study, which was not the case for any of the participants. The scores were always discussed and participants were instructed that they could stop the tests at any moment.

### DW-MRI acquisition

All participants underwent MRI examination on a 3-T Philips Intera system (Best, The Netherlands) with a 32-channel head coil. Single shot echo-planar images (EPI) with SENSE (parallel) MRI scans were obtained. Fifty-five contiguous slices were acquired in the axial plane with the following parameters: repetition time (TR) = 8872 ms, echo time (TE) = 2.5 ms, flip angle = 90°, field of view (FOV) = 240 × 240 x 137.5, voxel size = 2.5 x 2.5 x 2.5 mm, 60 non-collinear directions, b-value 1000 s/mm^2^, 2 nondiffusion-weighted images. The scan acquisition time was 13:52 min.

### DTI processing

Diffusion-weighted images were pre-processed using the software ExploreDTI (version 4.8.3) [66]. Images were corrected for subject motion and eddy current-induced distortions. The diffusion tensor model was applied and whole-brain tractography was conducted using the following stopping criteria 1) fractional anisotropy (FA-threshold) < .20 and 2) a turning angle between voxels > 40°. Step length between calculations was 1 mm. Afterwards, diffusion-weighted images were visually checked for subject motion in the scanner. A quantitative measure for head motion was calculated as the root mean square of the mean displacement in the three directions. In the present sample no participant met the criterion for excessive head motion, as defined by Power et al. [67] as RMS movement that exceeds 1.5 mm. However, the RMS of mean head movement was taken into account for statistical analyses (see section on Statistical analyses for more information).

The software TrackVis (trackvis.org) was used to perform virtual in vivo dissections applying deterministic tractography. Manual dissection has the advantage that brains can be analysed in native space, avoiding normalization artefacts. All white matter pathways, i.e. the three segments of the AF, the IFOF, the UF and the ILF (Fig 1) were dissected in the left hemisphere using a region of interest (ROI) approach with two obligatory passages, as described by Catani and Thiebaut de Schotten [68] and Wakana et al. [69]. The left UF was not traceable in five brains. All other pathways were present in all participants. For all white matter pathways, a mean fractional anisotropy (FA) index was extracted. The FA index, that is the most frequently applied index to quantify white matter organization, is an indirect measure of white matter organization that is driven by microstructural properties such as myelination and axon density, and macrostructural properties such as fiber crossings [70]. In addition, axial and radial diffusivity indices were extracted for each pathway, representing the magnitude of the diffusion in the principal direction and in the directions perpendicular to the principal direction, respectively.

### Statistical analyses

**Fig 2:**
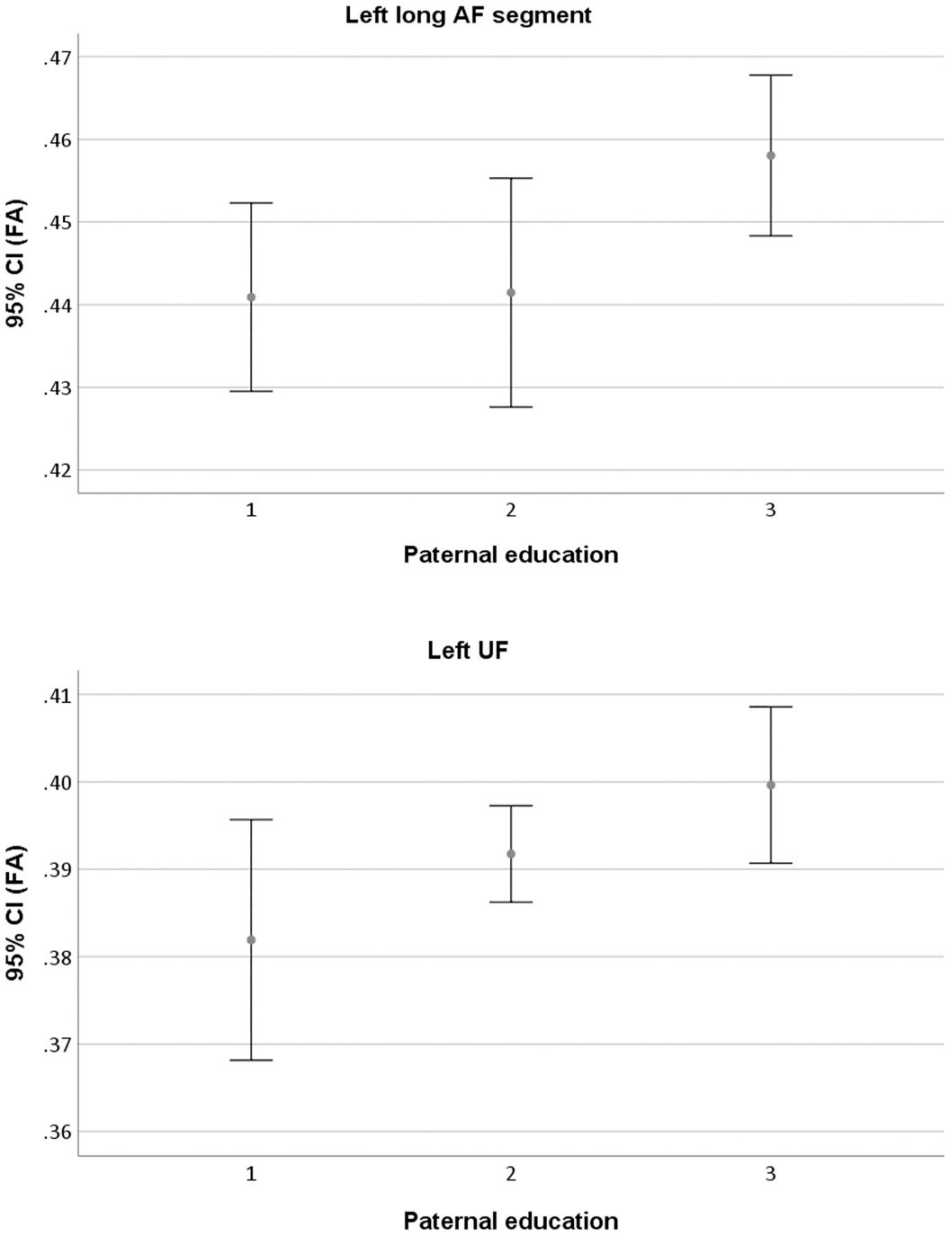
Schematic overview of the relation between environmental influences, quantified by paternal educational level, dorsal and ventral white matter structure and word reading of the offspring. Note that all arrows are bidirectional.

All statistical tests were performed at the two-tailed level (*p* < .05). After checking assumptions, general linear models were run to investigate the relation between SES as quantified by paternal educational level, reading and white matter structure. In the next sections, the different statistical analyses are described with more detail. Note that for each statistical model, the variables that are indicated on the left side of Fig 2 are applied as predictors for the variables on the right side of this figure. Nevertheless, relations should be interpreted bidirectionally as no causality can be determined. Finally, subject head motion, quantified by the RMS of mean head movement, was taken into account as a covariate variable in all statistical models containing FA of white matter pathways. However, removing this covariate from the statistical analyses did not change any of the results. To decrease the chance of over-correction and increment of type II errors, correction for multiple comparisons was only applied for post-hoc analyses. In addition, to decrease the chance of type I errors, a multivariate general linear model was executed in case multiple models with the same predictors were run.

#### Association between paternal educational level and reading

In a first step, the relation between paternal educational level and reading was investigated by two general linear models, predicting word reading and pseudo-word reading, respectively, with educational level of the father as predictor (three categories). Only reading measurements that were significantly related to paternal education, were included in the subsequent analyses.

#### Association between white matter structure and reading

A general linear model was applied for each left hemisphere white matter pathway, including FA of the pathway as predictor and word reading as dependent variable. Subject head motion in the scanner was added as a covariate. In case white matter FA in a specific pathway significantly predicted reading outcome, we further explored whether the observed relation was driven by axial diffusivity and/or radial diffusivity. Therefore, two general linear models were run, including word reading as the dependent variable and axial or radial diffusivity of the pathway as predictor, respectively. Again, subject head motion was included as a covariate. For these post-hoc analyses correction for multiple comparisons was applied by means of Bonferroni correction.

#### Association between paternal educational level and white matter

In the next section, the relation between left white matter FA and educational level of the father was investigated by general linear models, with FA of the pathway as dependent variable and paternal educational level as predictor. Again, subject head motion was included as covariate. Given that six models with the same predictors were run, an integrative multivariate general linear model was run to decrease the chance of type I errors. Again, significant findings for white matter FA were further investigated by exploring whether these relations were driven by axial diffusivity and/or radial diffusivity. For these general linear models, axial or radial diffusivity was included as the dependent variable, with paternal educational level as predictor and subject head motion as covariate.

#### Specificity of the results: The role of family risk and vocabulary knowledge

In the final section, we investigated whether the observed relations between paternal educational level, reading and white matter were specific for the paternal educational level, or could be (partly) driven by the family risk for dyslexia. Therefore, we re-ran all statistical analyses that revealed a significant relation to paternal educational level and reading or white matter FA by means of GLM models including family risk status (two levels) as predictor, subject head motion as covariate and either word reading, FA in the left long AF segment or FA in the UF as dependent variable.

In addition, we investigated whether the observed relations between paternal educational level, white matter and reading were specific to word reading, or could be generalized to broader language skills, quantified by vocabulary knowledge. Therefore, a GLM was run with vocabulary knowledge as dependent variable and either paternal educational level, FA in the left long AF segment or FA in the left UF as predictors. Similarly, subject head motion was added as a covariate.

## Results

Characteristics of the participants and their parents are presented in Table 1. As indicated in the methods section, statistical analyses were restricted to paternal educational level, given that more data were missing for the mothers (fathers: 0 missing, mothers: 4 missing) because more fathers were diagnosed with dyslexia compared to the mothers, and the educational level of the mothers was not equally distributed.

### Association between paternal educational level and reading

Given that the present study aims to investigate the relationship between parental educational level as measurement of SES, reading skills and structural organization of left white matter pathways, we first conducted an exploratory analysis to investigate whether we could replicate the previously reported relation between parental SES, in our sample represented by paternal educational level, and the offspring’s reading. The results revealed that paternal education level was indeed significantly related to word reading of the child (F_(2,31)_ = 3.721, *p* = .036). No relation existed between paternal educational level and pseudo-word reading (F_(2,31)_ = 2.240, *p* = .123). Given that interpretability of a potential relation between SES, reading and white matter measures would be hampered in case the previously demonstrated relation between SES and reading is not retrieved, the subsequent analyses are restricted to word reading skills as measures of reading ability of the offspring.

### Association between white matter structure and reading

Next, we explored the neural correlates of word reading within the adolescents. A general linear model was applied for each left hemisphere white matter pathway, including FA of the pathway as predictor and word reading as dependent variable. Subject head motion in the scanner was added as a covariate. For the dorsal white matter pathways, a significant relation to word reading was found for the left long AF (F_(1,31)_ = 5.078, *p* = .031). No significant relation was found between word reading and FA of the left anterior segment (F_(1,31)_ = .019, *p* = .294) or left posterior segment (F_(1,31)_ = .023, *p* = .227). For the ventral white matter pathways, a significant relation was found between word reading and FA of the left UF (F_(1,26)_ = 5.075, *p* = .033), whereas FA of the left IFOF (F_(1,31)_ = 2.242, *p* = .144) and FA of the left ILF (F_(1,31)_ = .389, *p* = .537) were not significantly related to word reading. Overall, no effect of subject head motion in the scanner was found (*p*_*s*_ > .40).

For FA in the two pathways that significantly predicted reading outcome, we further explored whether the observed relation with word reading was driven by axial diffusivity and/or radial diffusivity. The analyses revealed that the relation between FA of the left long AF and word reading was negatively driven by radial diffusivity (F_(1,31)_ = 8.200, *p* = .007), while no relation with axial diffusivity was observed (F_(1,31)_ = .758, *p* = .391). This effect of radial diffusivity survives Bonferroni correction for multiple comparisons. These results indicate that higher word reading scores are related to smaller radial diffusivity in the left long AF, i.e. smaller diffusivity in the directions perpendicular to the principal direction. For the left UF, similarly, the effect observed for FA of the left ventral UF seemed negatively driven by radial diffusivity (F_(1,26)_ = 5.306, *p* = .029), although this effect did not survive Bonferroni correction for multiple comparisons. No relation with axial diffusivity was observed (F_(1,26)_ = 1.508, *p* = .230). Again, no effect of subject head motion was found (*p*_*s*_ > .42).

### Association between paternal educational level and white matter structure

**Fig 3:**
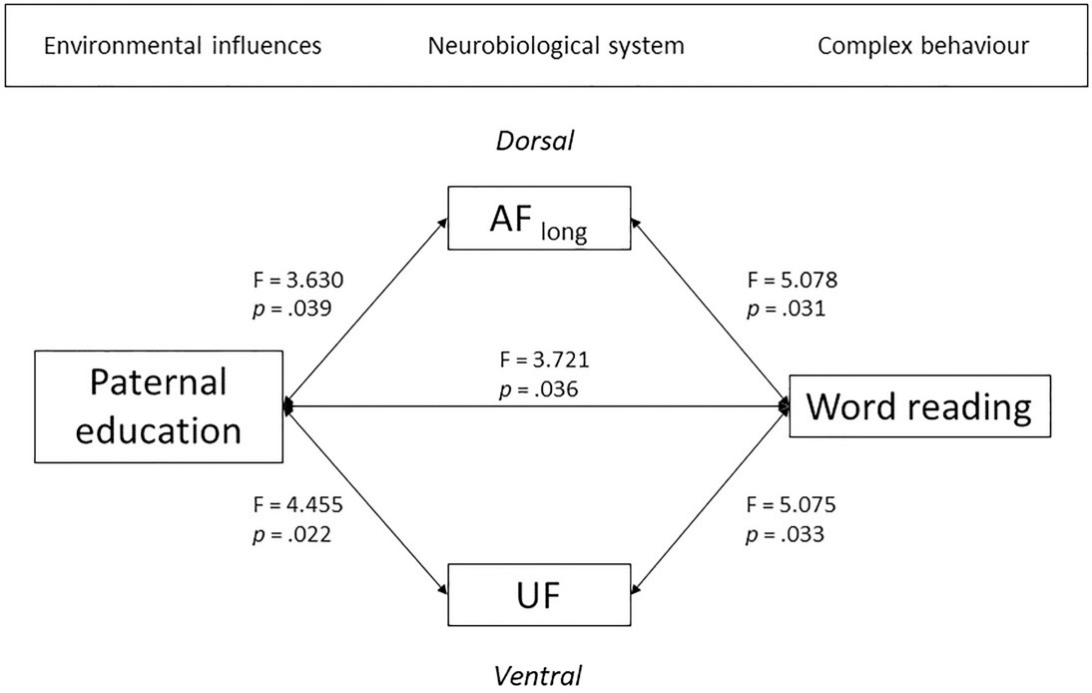
Visual representation of the relation between paternal educational level and FA of the left long AF segment (top panel) and FA of the left UF (bottom panel). The error bars represent the 95% confidence interval (CI).

In this section the relation between SES, quantified by educational level of the father, and FA of the different dorsal and ventral pathways of the left hemisphere was investigated. Therefore, a general linear model was run for each white matter pathway, with FA of the pathway as dependent variable, paternal educational level as predictor and subject head motion as covariate. Of specific interest was to determine whether FA of those pathways that was related to word reading, was also related to paternal educational level, thereby closing the circle. Indeed, a significant relation between paternal educational level and white matter FA was retrieved for both the left long AF segment (F_(2,31)_ = 3.630, *p* = .039) and the left UF (F_(2,25)_ = 4.455, *p* = .022) (see Fig 3). Again, no significant relation was observed between educational level of the father and FA of the left anterior AF segment (F_(2,31)_ = 2.365, *p* = .111), FA of the posterior AF segment (F_(2,31)_ = .403, *p* = .672), FA of the IFOF (F_(2,31)_ = .401, *p* = .673) and FA of the ILF (F_(2,31)_ = 1.313, *p* = .284). There was no effect of subject head motion (for the ILF: F_(1,30)_ = 4.079, *p* = .052; for the UF: F_(1,25)_ = 3.539, *p* = .072; for all other pathways: *ps* > .50). For a schematic overview of the main results, see Fig 2. The additional multivariate general linear model with paternal educational level as predictor, subject head motion as covariate and FA in the six white matter pathways as dependent variables confirmed the significant relation between paternal education for the left UF (F_(2,25)_ = 4.455, *p* = .022), whereas the relation for the left long AF segment did not reach significance anymore (F_(2,25)_ = 2.720, *p* = .085). There was again no relation between paternal educational level and FA of the left anterior AF (F_(2,25)_ = 1.004, *p* = .381), FA of the posterior AF (F_(2,25)_ = .011, *p* = .989), FA of the IFOF (F_(2,25)_ = 1.463, *p* = .251) and FA of the ILF (F_(2,25)_ = 1.930, *p* = .166).

For the left long AF and the left UF, we again further explored whether effects were driven by axial and/or radial diffusivity. A general linear model was run to predict either axial diffusivity or radial diffusivity of both pathways, independently. For the long AF segment, no relation was found between paternal educational level and either axial (F_(2,30)_ = .750, *p* = .750) or radial (F_(2,30)_ = 1.739, *p* = .193) diffusivity. Similarly, for the left UF, no significant relation was found for either axial (F_(2,25)_ = .632, *p* = .540) or radial diffusivity (F_(2,25)_ = 2.264, *p* = .125). There was no effect of subject head motion (*p*_*s*_ > .08).

### Specificity of the results: The role of family risk for dyslexia and vocabulary knowledge

In this section, we explored whether the observed relations between paternal educational level, word reading of the adolescents and white matter structure can be (partly) explained by family risk of the adolescents, as 18 adolescents had a family risk for dyslexia. Therefore, we re-ran all statistical analyses that revealed a significant relation to paternal educational level and reading or white matter FA. First, the relation between family risk for dyslexia and word reading was investigated. The results revealed no significant relation between word reading of the offspring and the family risk status (F_(1,31)_ = 2.126, *p* = .155). Next, the relations between family risk and FA of the long AF segment and FA of the ventral UF segment were investigated. Along the same lines, no relations were observed between family risk status and FA of the long AF segment (F_(1,31)_ = .098, *p* = .756) or FA of the left UF (F_(1,26)_ = 1.687, *p* = .205). Hence, the relation between paternal educational level, reading and white matter was not driven by the family risk status of the child.

Finally, we investigated whether the observed relations between paternal educational level, white matter and reading were specific to reading, or could be generalized to broader language skills, quantified by vocabulary knowledge. No relation between paternal education level and vocabulary knowledge existed (F_(2,31)_ = .133, *p* = .876). Similarly, no relation existed between vocabulary knowledge and white matter FA of the left long AF (F_(1,32)_ = 1.321, *p* = .259) or FA of the left UF (F_(1,27)_ = 2.060, *p* = .163).

## Discussion

The present study investigated the relation between socio-economic status (SES), reading and structural organization of dorsal and ventral white matter pathways of the left hemispherical reading network. The results revealed a relation between SES, defined by paternal educational level, word reading and white matter structure, as quantified by FA, for the dorsal long arcuate fasciculus (AF) segment and the ventral uncinate fasciculus (UF) segment. The associations with paternal educational level were most robustly found in the ventral UF. The present study thereby confirms the relation between SES of the family and reading of the offspring, and expands the previously reported relation between SES, reading and white matter structure [51] by allocating the relation to specific dorsal and ventral white matter pathways, i.e. the left long AF and the UF, respectively.

Environmental influences on reading have been demonstrated multiple times (e.g. [6,17,21,22]) and these influences have been acknowledged in recent theories on developmental dyslexia, e.g. the multiple deficit model by Pennington [26] and the intergenerational multiple deficit model by van Bergen et al. [71]. However, the neural correlates of these environmental influences have been less thoroughly investigated. Interestingly, in the present study, we observed a relation between SES and white matter structure in pathways that are associated with reading ability. Hence, a relation between paternal educational level and word reading of the adolescent was observed, as well as a relation between paternal educational level and FA of one ventral white matter pathway and one dorsal white matter pathway, although the latter did not reach significance in a multivariate general linear model. Finally, FA of the same dorsal and ventral white matter pathways was related to word reading of the adolescents.

The left dorsal pathway, i.e. the left long AF segment, is the segment within the left AF that has been most related to reading and cognitive abilities underlying reading. The left long AF segment has been related to phonological processing in pre-readers [72–73] and in adults [49], and lower FA has been demonstrated in adults with developmental dyslexia [49]. This difference in FA abnormalities has been reported, as well, in pre-readers at cognitive risk for dyslexia [72] and has recently been confirmed to precede reading acquisition in children who developed poor reading skills later on [47–48]. Although the study by Gullick et al. [51] did not include tractography, they did attribute the cluster where a relation between education level of the parents and reading was observed to the left long AF segment. No relation with parental education and/or reading was observed in the left anterior and posterior segments of the AF. The absence of a relation between reading and FA of the left anterior AF is not unexpected, as the anterior AF segment has been related to reading less often. A recent study demonstrated, however, an increase in FA of the left anterior AF segment in children who could read, relative to age-matched pre-readers [74]. Moreover, they reported a relation between FA of the left anterior AF and phonological processing. FA of the left posterior AF segment has been reported to increase when ex-illiterates learned to read [75], and has been related to phonological aspects of reading in pre-readers [76]. However, the pathway was not related to word reading in our sample of adolescents and no relation between paternal educational level and FA of these white matter pathways was found, either.

Within the left ventral white matter network, a relation was observed between paternal educational level, reading and FA of the UF. The role of the UF in reading has only recently been reported. FA of the left UF has been related to reading in adults [77,78], whereas in children FA of the right UF has been demonstrated to be related to reading ability [74,79]. Vanderauwera et al. [80], showed that the relation between reading-related cognitive skills and FA of the left IFOF and right UF developed in a similar vein throughout the first two years of reading acquisition, demonstrating a relation to orthographic knowledge at an early reading stage. However, while our results suggest that FA of the UF is related to the paternal education level in a sample of adolescents, a longitudinal study in young children demonstrated that FA of the left IFOF deviated in young children with a familial risk for dyslexia relative to children without a family risk [80]. In addition, in the same children, FA of the IFOF has also been demonstrated to be related to self-reported reading skills of the father and to a measurement of SES driven by family income [81]. In the present study, the relation between paternal educational level and FA of both the UF and the left long AF was not driven by having a family risk for dyslexia. Hence, FA of the UF and the long segment of the AF seem to be more closely related to educational level of the father, whereas FA of the IFOF seems more closely related to familial risk of dyslexia, parental reading level and family income. Hence, we hypothesize that paternal educational level might be related to the provided home literacy environment, and operates in this way as a purer environmental influence compared to the family risk status, that is also driven by genetic factors. At a young age, when children have no reading experience, the home literacy environment might play a smaller role than at the adolescent age. Vandermosten et al. [81] indeed demonstrated no effect of the home literacy environment on cognitive skills and on white matter FA at the pre-reading level. However, this hypothesis needs to be further investigated. Finally, no relation between FA of the left ILF, reading and/or SES has been found in the present study. A relation of FA of this structure with cognitive aspects of reading has been suggested by Yeatman et al. [82], although these results were not consistently observed [72,78]. Gullick et al. [51] did report a relation between SES and white matter structure in clusters attributed to bilateral ILF. However, the anatomical location of the reported clusters is in close proximity to the UF pathway.

We investigated whether the observed relations between reading, SES and white matter structure are specific for reading or could be retrieved for broader language skills, as well. The results indicated that the observed findings for reading could not be retrieved between broader language skills, as quantified by vocabulary knowledge, and white matter structure, nor between broader language skills and SES. Hence, the observed relation was specific to reading.

Although this study expands current literature by demonstrating interesting findings on the relation between SES, white matter and reading, some restrictions and specifications of the present study should be considered. First, in the present study SES is defined by the educational level of the father as educational level of the mother was missing for four individuals and data were not equally distributed. However, previous studies investigating the relation between SES, brain structure and reading included the SES of the mother [50] or of both parents [43,51]. In addition, we withdrew from conducting mediation analysis as we applied a categorical variable for paternal educational level. Applying a more detailed measurement is advised to enable mediation analyses. Second, we explored whether axial and/or radial diffusivity drove the observed relations for white matter FA. The relations between word reading and FA of the left long AF and UF were negatively driven by radial diffusivity, i.e. the diffusivity in the direction perpendicular to the direction of the principal eigenvector [70]. For the relation between paternal educational level and FA of the left long AF and UF, no clear pattern could be observed for axial or radial diffusivity. The interpretation of this index is not straightforward and directly relating the index to underlying microstructural and macrostructural properties should be done with extreme caution [83]. Hence, for an in-depth interpretation of the specific contribution of underlying micro-structural and macro-structural properties on the observed effect in FA and radial diffusivity, future research is required, combining tractography of individual tracts with microscopic quantitative measurements. This approach can enhance our understanding of microscopic white matter structure and macrostructural influences [84]. Third, although the present study included *in vivo* tractography of six white matter pathways in the left hemisphere, future research should include the investigation of the relation between parental education level, word reading and structural organization of other pathways that have been related to reading and dyslexia, such as the thalamic radiations [85–88]. Finally, the relation between paternal educational level and reading was restricted to word reading, and no relation was found with pseudo-word reading. At the adolescent age, and in a relatively transparent language such as Dutch, individuals are thought to attain more strongly to the lexical reading route for reading over the sublexical grapheme-phoneme decoding strategy. Hence, this might explain why the observed relations were restricted to word reading. However, the reading measures used in the present study cannot clearly disentangle different reading strategies. We therefore suggest that further research should focus on investigating the relation between different reading strategies, parental educational level, and if a relation is retrieved, also with structural organization of white matter pathways. In the future, the latter step ideally includes more fine-grained measures of underlying microstructural properties.

In sum, the present study expands current insight in the relation between SES, reading and white matter structure by attributing the previously reported relation with white matter to specific dorsal and ventral white matter pathways in the left hemisphere, i.e. the dorsal long AF and the ventral UF. Further research is, however, required to define the underlying factors that drive the specific measurements of SES, and to determine the specific contribution of environmental and genetic influences on brain structure and functioning of the children.
